# Bibloc lombaire et sciatique plexique pour la chirurgie urgente des fractures pertrochantériennes: une technique alternative chez les patients à haut risque anesthésique

**DOI:** 10.11604/pamj.2020.37.12.21392

**Published:** 2020-09-03

**Authors:** Ismail Aissa, Loukman El Wartiti, Najib Bouhaba, Said Khallikane, Mohamed Moutaoukil, Noureddine Kartite, Abdelghafour Elkoundi, Aziz Benakrout, Abdellatif Chlouchi, Anas Elbouti, Hamza Najout, Ali Grine, Reda Touab, Abderrahim Zaizi, Jalal Youssef, Hicham Bakkali, Hicham Balkhi, Mustapha Bensghir

**Affiliations:** 1 Pôle d’Anesthésie-Réanimation, Hôpital Militaire d’Instruction Mohamed V, Faculté de Médecine et de Pharmacie, Université Mohamed V, Rabat, Maroc,; 2 Service de Traumatologie Orthopédie, Hôpital Militaire d’Instruction Mohamed V, Faculté de Médecine et de Pharmacie, Université Mohamed V, Rabat, Maroc

**Keywords:** Bibloc lombaire et sciatique, chirurgie urgente, fracture pertrochantérienne, patients à haut risque anesthésique, Lumbar plexus-sciatic nerve block, emergency surgery, pertrochanteric femoral fractures, patients at high risk of anaesthetic complications

## Abstract

**Introduction:**

l’anesthésie pour la chirurgie urgente de la fracture pertrochantérienne (FPT) chez les patients à haut risque anesthésique représente souvent un véritable challenge pour les praticiens en vue du risque periopératoire majeur. Nous rapportons notre expérience avec le bibloc ou bloc combiné lombaire et sciatique plexique (BCLS) comme technique anesthésique alternative face à ce type de situation.

**Méthodes:**

une étude transversale, descriptive, monocentrique, a été menée sur une période de 3 ans, incluant les patients à haut risque anesthésique présentant une FPT récente. Les deux blocs nerveux étaient réalisés au niveau plexique selon la technique classique de neurostimulation. Un mélange de 20ml de lidocaine 2% et de bupivacaine 0,5% (50/50) a été injecté au niveau de chaque bloc. Le critère d’évaluation principal était l’efficacité du BCLS apprécié par l’incidence d’échecs de la technique anesthésique, définie par la nécessité de convertir en anesthésie générale (AG). Les critères d’évaluation secondaires étaient: 1) les données techniques de la procédure anesthésique, 2) les retentissements hémodynamiques, respiratoires et neurologiques periopératoires, et 3) les résultats et les complications éventuelles en postopératoire.

**Résultats:**

trente patients ont été colligés. L’âge moyen était de 74 ± 10 ans. Le délai moyen d’admission aux urgences-intervention était de 12(5-36) heures. La durée moyenne pour la réalisation de la procédure était de 15,20 ± 3,45 minutes. Aucune conversion en AG n’a été nécessaire. Il n’y avait pas de différences statistiquement significatives entre les différentes valeurs enregistrées des paramètres hémodynamiques et respiratoires periopératoires (PAM, FC, SpO2) (p > 0,05). La durée de l’intervention était de 46 ± 5 minutes. La satisfaction des chirurgiens était de 9,7 ± 0,1. La première demande en antalgiques postopératoires était après 8(1-24) heures. Tous les patients avaient une récupération sensitivomotrice complète.

**Conclusion:**

le BCLS est une alternative anesthésique pour les interventions urgentes de FPT chez les patients à haut risque anesthésique: délais opératoires réduits, efficacité anesthésique, stabilité hémodynamique et respiratoire periopératoire, absence de complications inhérentes aux autres techniques anesthésiques, passage rapide en salle de surveillance post-interventionnelle (SSPI), et analgésie postopératoire de qualité.

## Abstract (en)

**Introduction:**

emergency surgery for pertrochanteric femoral fractures (PFF) in patients at high risk of anaesthetic complications is a real challenge for surgeons due to the increased intraoperative risk. We report our experience with combined lumbar plexus-sciatic nerve block as an alternative anesthetic technique for these fractures.

**Methods:**

we conducted a three-year descriptive, single-center, cross-sectional study including patients with a history of recent pertrochanteric femoral fractures (PFF) at high risk anaesthetic complications. Combined lumbar plexus-sciatic nerve block was performed using the common neurostimulation technique. A mixture of 20ml of lidocaine 2% and bupivacaine 0.5% (50/50) was injected into each block. The primary endpoint was the effectiveness of lumbar plexus-sciatic nerve block assessed through the rates from anesthesia-related failures defined as need for conversion into general anaesthesia (GA). The secondary endpoints were: 1) anesthetic technique, 2) intraoperative hemodynamic, respiratory and neurological impairment, and 3) outcomes and potential postoperative complications.

**Results:**

the study included 30 patients. The average age of patients was 74 ± 10 years. The average admission time in the Department of Emergency Surgery was 12(5-36) hours. The average duration of the procedure was 15.20 ± 3.45 minutes. No conversion into GA was necessary. There were no statistically significant differences between the various recorded intraoperative hemodynamic and respiratory parameters (MAP, HR, SpO2) (p > 0,05). Surgical procedure duration was 46 ± 5 minutes. Surgical satisfaction was 9.7 ± 0.1. The first post-operative analgesic treatment was started after 8(1-24) hours. All patients had complete sensorimotor recovery.

**Conclusion:**

combined lumbar plexus-sciatic nerve block is an anesthetic alternative for urgent PFF surgery in patients at high risk of anaesthetic complications: reduced operative delays, anesthetic efficiency, hemodynamic and intraoperative respiratory stability, absence of complications due to other anesthetic techniques, rapid admission to recovery room, and good postoperative analgesia.

## Introduction

Les fractures per trochantériennes (FPT) représentent près de 50% des fractures de l’extrémité supérieure du fémur [1]. Elles constituent l’un des principaux problèmes de santé à travers le monde. Leur incidence est en augmentation progressive due au vieillissement de la population et à l’incidence élevée de l’ostéoporose chez les personnes âgées [2]. Ces fractures sont responsables d’une morbi-mortalité considérable en particuliers chez les patients âgés avec lourdes comorbidités [3, 4]. L’amélioration du pronostic associé à ces lésions passe avant tout par une fixation sans retard de la fracture permettant un lever précoce avec réinsertion sociale. L’ensemble des études s’accordent sur un délai < 24-72h entre le traumatisme et la chirurgie [5]. Les patients avec comorbidités lourdes classés *American Society of Anesthesiologists* (ASA) III et IV représentent un véritable challenge anesthésique en vue du risque peri-opératoire majeur. Les études s’accordent sur les avantages de l’anesthésie perimédullaire (APM) en particulier la rachianesthésie (RA) par rapport à l’anesthésie générale (AG) pour les interventions de fracture de la hanche (FH) en termes de réduction significative de la morbi-mortalité [6-8].

Néanmoins, chez certains patients avec comorbidités lourdes l’APM pourrait ne pas être appropriée en raison des risques associés d’hypotensions peropératoires, de nausées-vomissements, de rétentions urinaires, d’hématome peripidural etc. [9]. Récemment, le bibloc ou bloc combiné lombaire et sciatique (BCLS) a été introduit comme une nouvelle méthode anesthésique en chirurgie de FH avec une efficacité comparable à celle de la RA [10]. A travers des cas isolés ou des séries de faibles effectifs le BCLS a été pratiqué efficacement chez des patients fragiles à haut risque anesthésique [11-15]. Néanmoins, des études à larges effectifs explorant le BCLS chez ce type de patients sont très rares. Il reste de pratique moins courante par rapport à l’APM ou l’AG principalement en raison des délais et de l’expertise requises pour sa réalisation [16]. L’objectif de notre étude était d’évaluer l’efficacité, la sécurité, et les effets adverses associés au bloc lombaire plexique (BLP) combiné au bloc sciatique plexique (BSP) ou BCLS pour les interventions urgentes de fixation des FPT chez les patients graves à haut risque anesthésique.

## Méthodes

**Patients, équipe anesthésique et chirurgicale:** après accord du comité d’éthique de l’Hôpital Militaire d’Instruction Mohamed V de Rabat, une étude transversale, descriptive, a été menée au bloc des urgences sur une période de 3 ans de juin 2015 à juillet 2017. Un consentement éclairé et signé était recueilli pour chaque patient. Les critères d’inclusions étaient les patients adultes à haut risque anesthésique définis par un score ASA à III-IV présentant une FPT récente. Les critères d’exclusion étaient: le refus de participer à l’étude, les patients ASA I-II, les patients avec fractures multiples, neuropathie périphérique, trouble de l’hémostase, patients sous anticoagulants ou deux antiagrégants plaquettaires, infections aux points de ponction et allergie aux anesthésiques locaux (AL). L’équipe chirurgicale comprenait un senior expérimenté aux FPT, un médecin résidant et un aide paramédical. Tous les blocs nerveux étaient réalisés par le premier auteur qui était un médecin anesthésiste avec une expertise en anesthésie loco-régionale perinerveuse (ALR-PN) avec l’aide d’un infirmier anesthésiste qualifié.

**Déroulement de l’anesthésie perinerveuse:** au bloc opératoire les patients ont bénéficié d’un monitorage incluant pression artérielle (invasive ou non invasive en fonction du statut cardiovasculaire), électrocardiogramme (scope à cinq brins avec analyse du segment ST), et saturation pulsée en oxygène (SpO2). Une perfusion douce d’un soluté de sérum isotonique était réalisée (200ml). Une sédation de confort à base du midazolam (0,025mg/kg) ± fentanyl (25-50ug) était apportée sous oxygène nasal (2 L/min). L’antibioprophylaxie était à base de cefazoline (2g). Les patients étaient installés soigneusement en décubitus latéral (côté fracturé en haut), hanches et épaules maintenus parallèles, tête fléchie, lordose lombaire réduite, et le membre à opérer en légère flexion de la hanche et du genou.

Une procédure identique était utilisée pour les deux blocs nerveux chez tous les patients. Après désinfection du point de ponction et réalisation d’une anesthésie locale, le repérage nerveux était fait par la technique classique de neurostimulation à l’aide d’une aiguille à biseau court 22-gauge (BBraun, Melsungen, Allemagne) d’une longueur de 100mm (150mm chez l’obèse) connectée à un neurostimulateur (HNS12, BBraun, Melsungen, Allemagne) dont l’intensité de stimulation, la fréquence et la durée de stimulation étaient respectivement réglées à 2mA, 1Hz et 0,1ms. Après obtention de la réponse motrice recherchée la position de l’aiguille était optimisée de façon à conserver une réponse motrice pour une intensité minimale de stimulation comprise entre 0,4-0,5mA. A cette intensité, et après un test aspiratif, une injection fractionnée des AL était réalisée avec un mélange de lidocaine 2% et de bupivacaine 0,5% (avec un rapport volume de 1/1). Les volumes injectés étaient de 20ml au niveau lombaire, et 20ml au niveau sciatique.

Les deux blocs plexiques étaient réalisés dans un même ordre chronologique pour tous les patients: lombaire puis sciatique. Le BLP était réalisé selon l’approche décrite par Capdevila *et al*. [17]; on trace une ligne horizontale unissant le sommet des crêtes iliaques, puis on trace une ligne verticale passant par les apophyses épineuses et finalement une ligne parallèle à la ligne unissant les épineuses et passant par l’épine iliaque postérieure et supérieure (EIPS). Le point de ponction se situe à l’union du tiers latéral et des deux tiers médiaux de la perpendiculaire à la droite passant par l’EIPS rejoignant l’apophyse épineuse de L4, soit environ 4cm en dehors de l’apophyse épineuse de L4. L’aiguille était avancée perpendiculairement à la peau jusqu’au contact avec le processus transverse de L4 puis ensuite retirée de 0,2cm et avancé de 1 à 2cm au-dessous du processus transverse jusqu’à l’obtention des contractions du muscle quadriceps (ascension de la rotule).

Le BSP était réalisé par approche para-sacrée selon la technique de Mansour [18]; en gardant le patient dans la même position une ligne était tracée entre l’EIPS et la tubérosité ischiatique, le point de ponction se trouve sur cette droite 6cm au-dessous de l’EIPS. La même aiguille était introduite perpendiculairement à tous les plans jusqu’à l’obtention d’une flexion plantaire ou une dorsiflexion du pied. L’installation du bloc sensitif était évaluée par pinprick test (0 = pas de bloc, 1 = sensibilité partielle, 2 = insensibilité complète), et pour le bloc moteur par le score de bromage (0 = pas de bloc moteur; 1 = hanche bloquée; 2 = hanche et genou bloqués; et 3 = hanche, genou et cheville bloqués) [19]. Une extension du bloc sensitif et/ou moteur était également recherchée dans l’autre membre inférieur (surtout pour le BLP). L’évaluation de l’installation des blocs nerveux était réalisée à partir de la fin de la dernière injection par intervalles de 5 minutes.

**Périodes per et postopératoires:** une fois les patients étaient sans douleur, ils étaient placés en décubitus dorsal puis une réduction fermée de la fracture était réalisée. L’incision chirurgicale était autorisée lorsque les blocs sensitifs et moteurs étaient complets. En l’absence d’installation de ces blocs après 40 minutes une conversion en AG était prévue. Les paramètres hémodynamiques (FC, pression artérielle moyenne, rythme cardiaque, segment ST), respiratoires (fréquence respiratoire, SpO2) et neurologiques (contact verbal permanent) étaient régulièrement surveillés (toutes les 3 minutes) jusqu’à l’incision, puis toutes les 5 minutes jusqu’à la fin de l’intervention chirurgicale. L’éphédrine était administrée selon un protocole standard de 3-6mg lorsque la TA systolique était < 90mmHg, ou lorsque la diminution de la TA de base était > 30%.

En postopératoire, les patients étaient admis en salle de surveillance post-interventionnelle (SSPI) où l’évaluation de la douleur était réalisée selon l’échelle visuelle analogique, EVA de 0 à 10 (0 = absence de douleur et 10 = la douleur la plus intense ressentie). Si nécessaire, une analgésie intraveineuse additionnelle était prévue à base du paracétamol 1g ± nefopam 20mg et/ou morphine 2-3mg jusqu’à l’obtention d’un score EVA inférieur à 3/10. En absence de complications les patients quittaient la SSPI lorsque le score d’Aldrete était supérieur à 12/14 soit vers le service de traumatologie soit vers l’unité de soins intensifs (USI) en fonction des comorbidités associées et du déroulement opératoire. Au service d’accueil un monitorage standard était mis en place pour tous les patients, des visites régulières étaient assurées par l’infirmier soignant avec évaluation régulière de l’EVA, du premier besoin en antalgiques et de tous les effets adverses possibles. Le statut neurologique du membre opéré était évalué régulièrement par le chirurgien au cours des visites postopératoires. La présence de dysesthésies ou d’autres troubles neurologiques étaient signalés au médecin anesthésiste.

### Collecte des données

***Les données préopératoires:*** l’âge, le sexe, le poids, la taille, les comorbidités associées, les traitements en cours, le statut ASA, les délais fracture-admission aux urgences et admission aux urgences-intervention.

***Au bloc opératoire:*** la durée moyenne de réalisation des blocs nerveux (début de ponction-fin de la dernière injection), les incidents liées à la procédure anesthésique (ponction vasculaire, difficulté ou douleur à l’injection, hypotension après la procédure…), le délai d’installation des blocs, le confort des patients pendant la réalisation des blocs et durant l’intervention, le délai réalisation des blocs-début d’installation chirurgicale, la durée de l’installation chirurgicale, les incidents peropératoires (hémodynamiques, respiratoires, neurologiques, saignement, douleur et besoins en antalgiques…), la technique chirurgicale et la durée de l’intervention. La satisfaction du chirurgien quant au degré de l’immobilisation et du relâchement musculaire du site opératoire était notée en utilisant une échelle de 0 à 10 (0: intervention impossible, 10: conditions opératoires idéales).

***Les données postopératoires:*** complications postopératoires immédiates liées à l’anesthésie, besoins en USI postopératoires, durée de séjour en USI, le premier besoin en antalgiques, les antalgiques utilisés et les complications neurologiques tardives liées aux blocs nerveux. La satisfaction des patients par rapport à la technique anesthésique était évaluée subjectivement par le souhait (ou non) d’avoir la même anesthésie pour une prochaine intervention.

**L’analyse statistique:** les données ont été collectées, codées, et analysées à l’aide du logiciel SPSS pour Windows, Version 18 (SPSS, Inc, Chicago, IL, USA). Les variables quantitatives étaient exprimées en moyennes et déviations standards (m ± DS) si elles avaient une distribution normale (selon le test de kolmogorov-smirnov). Dans le cas contraire, elles étaient exprimées en médianes assortie des 25^e^ et 75^e^ percentiles. Les variables qualitatives étaient exprimées par leurs effectifs et leurs pourcentages (n,%). La corrélation entre la durée de réalisation de la procédure anesthésique et l’IMC et la corrélation entre pertes sanguines et prise d’antiagrégants plaquettaires ont étaient analysées avec le test de corrélation de Pearson, après s’être assuré de la distribution normale des valeurs. Pour la comparaison des différentes valeurs hémodynamiques et respiratoires (PAM, FC, SpO2) enregistrées à des temps différents en periopératoire, une analyse de variance pour mesures répétées a été appliquée. Une correction de Bonferroni a été appliquée sur ces tests en cas de significativité. Le seuil de signification retenu était p < 0,05.

## Résultats

Durant la période d’étude 30 patients ASA III-IV présentant une FPT récente ont été colligés (13 femmes et 17 hommes). Les caractéristiques démographiques sont exposées dans le Tableau 1. Les patients présentaient des comorbidités lourdes avec une ou plusieurs affections fragilisantes essentiellement de type cardiovasculaire et respiratoire (Tableau 1). Une polimédication était souvent observée. Le statut ASA était à III pour 21 patients (70%) et VI pour 9 patients (30%) (Tableau 1). Le délai moyen entre le traumatisme et l’admission aux urgences était généralement de 4 (2-18) heures, celui entre l’admission aux urgences et l’intervention était de 12 (5-36) heures (Tableau 2). Tous les patients ont reçu du midazolam + fentanyl (sauf 4 patients qui étaient hypoxémiques).

**Tableau 1:** caractéristiques sociodémographiques et cliniques de la population étudiée

		(n= 30)
Age (ans)^*^		74,25±10,16
**Sexe (H/F) (n)**		17/13
Poids (kg)^*^		77,88±17,25
IMC (kg/m^2^)^*^		26,43±3,57
**ASA#**	III	21(70)
	IV	9(30)
**Comorbidités#**	Insuffisance cardiaque	8(26)
	Valvulopathies	3(10)
	Cardiopathie ischémique	7(23)
	Insuffisance respiratoire	4(13)
	Insuffisance rénale	6(20)
	Hypertension artérielle	19(63)
	Diabète	18(60)
	Arythmie	8(26)
	Obésité	7(23)
	AVCI	3(10)
	Pneumonie	4(13)
	Asthme	4(13)
**Traitements poursuivis#**	Insuline	16(53)
	Antidiabétiques oraux	12(40)
	Bêtabloquants	13(43)
	Aspirine	16(53)
	Clopidogrel	4(13)
	IEC/ARAII	14(46)/12(40)
	Inhibiteurs calciques	15(50)
	Corticothérapie orale	4(13)

* Valeur exprimée en moyenne±écart-type#Valeur exprimée en effectif (pourcentage) ASA: American Society of anesthesiologists, AVCI: accidents vasculaires cérébrales ischémiques. IEC : inhibiteurs de l’enzyme de conversion, ARAII : antagonistes des récepteurs de l’angiotensine II

**Tableau 2:** données periopératoires

		(n= 30)
**Délais**	Traumatisme-admission aux urgences (h) #	4(2-18)
	Admission aux urgences-intervention (h)^*^	12 (5-36)
**Durées**	Réalisation des deux blocs (min)^*^	15,20 ± 3,45
	Réalisation des blocs-installation chirurgicale (min)^*^	10,75 ± 2,38
	Installation chirurgicale (min)^*^	32,94 ± 4,54
	Installation des blocs (min) ^*^	20,37 ± 3,43
	Intervention (incision-fermeture cutanée) (min) ^*^	40,95 ± 5,87
	SSPI (min)#	30 (15-60)
**Incidents durant le BCLS (n)**	Ponction vasculaire	2(6)
	Douleur à l’injection	1(3)
**Complications peropératoires (n)**	Hypotension marquée	2(6)
	Agitation	1(3)
**Saignement chirurgical (ml) #**		200(100-700)
**Unités globulaires transfusées (n) #**		0,5(0-3)
**Solutés perfusés (ml)#**		500(400-2000)
**Besoin en USI (n)**		13(43)
**Complications postopératoires précoces (<24h) (n)**	Acidocétose diabétique	1(3)
	NVPO	2(6)
**Durée de séjour à l’hôpital (j)#**		7(4-60)
**Complications postopératoires tardives (24h < 30j) (n)**	Infection respiratoire	1(3)
	Infection urinaire	1(3)
	Escarres lombo-sacrés	1(3)
	décès	1(3)

* Valeur exprimée en moyenne ± écart-type, #Valeur exprimée en médiane (25-75 %), Valeur exprimée en effectif (pourcentage). NVPO : nausées-vomissements postopératoires

La durée moyenne pour la réalisation des blocs était de 15,20 ± 3,45 (8-25) minutes. Une corrélation positive a été retrouvée entre les délais prolongés (> 20 minutes) et l’indice de masse corporelle (IMC) > 30kg/m^2^ (P < 0,05). Les incidents observés durant la procédure anesthésique étaient: 2 cas (6,6%) de ponction vasculaire et 1 cas (3,3%) de douleurs à l’injection durant le BSP résolus après correction de la position de l’aiguille. Aucun cas d’échec n’a été noté et par conséquence aucune conversion en AG n’a été nécessaire. 4 patients (13,3%) ont exprimé leur inconfort pendant la réalisation des blocs nerveux surtout à cause des mouvements du membre induits par la neurostimulation qualifiés de désagréables et douloureux à cause de la fracture. Le bloc lombaire était bilatéral chez 1 patient (3,3%) (partiel du coté sain/bloc moteur = 1). Deux patients (6,6%) ont présenté une hypotension artérielle prononcée, le premier 10 minutes après la fin des 2 blocs nerveux et le deuxième 5 minutes après le BLP (au cours du BSP), ayant nécessité respectivement 9mg et 15mg d’éphédrine. Un seul cas (3,3%) d’agitation brève a été noté chez un patient de 89 ans ayant nécessité une sédation continue au propofol. Le délai entre la réalisation des blocs et le début de l’installation chirurgicale était de 10 ± 2 minutes, la durée de l’installation chirurgicale était de 35 ± 4 minutes (Tableau 2).

**Figure 1:**
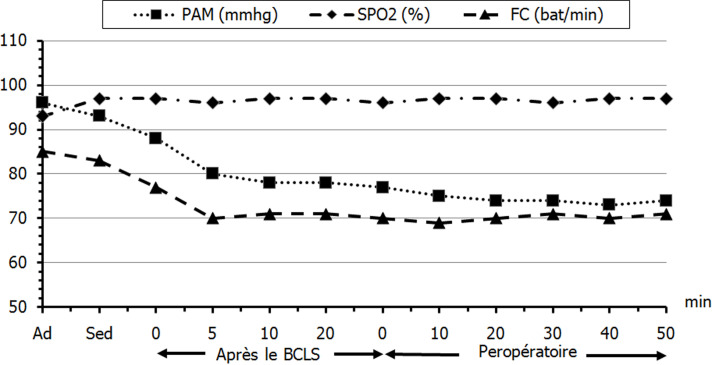
évolution des paramètres hémodynamiques et respiratoires periopératoires; (PAM: pression artérielle moyenne, FC: fréquence cardiaque, SPO2: saturation pulsée en oxygène, Ad: admission au bloc opératoire, Sed: sédation)

Les données hémodynamiques et respiratoires peropératoires sont exprimées dans la Figure 1. Il n’y avait pas de différences statistiquement significatives entre les différentes valeurs enregistrées des paramètres hémodynamiques et respiratoires periopératoires (PAM, FC, SpO2) (p > 0,05). Trois patients (10%) ont exprimé leur inconfort durant l’intervention à cause de la position chirurgicale et qui ont nécessité également une sédation continue au propofol. Aucune supplémentation antalgique peropératoire n’a été requise. La durée de l’intervention était de 46 ± 5 minutes (40-130). Tous les patients ont bénéficié d’une ostéosynthèse par un clou de type Gamma à foyer fermé. Le saignement chirurgical et le nombre de culots globulaires transfusés étaient respectivement de 200 ± 50ml (100-700) et 0.5 unités (0-3). Il n’y avait pas de différence statistiquement significative en termes de perte sanguine entre les patients avec et sans antiagrégants plaquettaires (p > 0,05). La quantité moyenne de solutés perfusés été de 500ml de sérum isotonique (400-2000).

La satisfaction des chirurgiens était évaluée à 10 pour 22 patients (73,3%) et à 9 pour 8 patients (26%). L’évaluation de la douleur postopératoire à la SSPI a objectivée un EVA ≤ 3 chez 26 patients (86%), n’ayant reçu aucune analgésie additionnelle, 4 patients (13%) avait 3 < EVA ≤ 5, dont 3 ont nécessité 1g de paracétamol seulement et le 4^e^ a nécessité 1g de paracétamol + 20mg de nefopam pour atteindre un EVA < 3. 13 patients (43%) ont été hospitalisé en USI vu leurs conditions médicales (tous transférés en service de traumatologie après 24 heures). La première demande en antalgiques postopératoires était généralement après 8 heures (1-24). 24 patients (80%) n’avaient besoin que d’antalgiques simples comme le paracétamol et/ou le nefopam, 6 patients (20%) ont nécessité en plus le recours à la morphine pour soulager la douleur (dose moyenne de 6 ± 2mg/24 heures). Sur 24 heures de surveillance rapprochée, 2 patients (6,6%) ont présenté des nausées et vomissements postopératoires (NVPO) attribuées aux morphiniques (traités avec succès par une seule dose de 4mg IV d’ondansétron). En dehors d’un cas de décompensation acido-cetosique chez une patiente diabétique (jugulée dans les 24 heures postoperatoires), aucun autre incident d’aggravation ou de décompensation des commorbidités sous-jacentes n’a été enregistré. Tous les patients avaient une récupération sensitivomotrice complète. Une hémoglobine était demandée en postopératoire pour cinq patients (16,6%) dont un seul (3,3%) a nécessité une transfusion de 2 culots globulaires. La durée moyenne de séjour à l’hôpital était de 7 jours (4-60). Vingt-six patients (86,6%) ont déclaré subjectivement qu’ils étaient satisfaits de leur choix de la technique anesthésique exprimé par le souhait d’avoir la même anesthésie pour une prochaine intervention.

## Discussion

La RA a été depuis longtemps privilégiée par la plupart des opérateurs comme technique anesthésique de choix pour les interventions de FH, car elle concilie les impératifs chirurgicaux et anesthésiques: technique efficace, de réalisation simple et rapide, reproductible et peu onéreuse. Néanmoins, le BCLS a été introduit comme alternative anesthésique pour ces interventions en particulier pour les patients à haut risque anesthésique [11-15, 20, 21]. Le BCLS procure une anesthésie unilatérale complète de la hanche du fait que l’innervation sensitivomotrice de cette région provient des rameaux nerveux issus des plexus lombaire et sciatique [10, 22]. Cette étude démontre l’intérêt du BCLS comme technique anesthésique efficace pour les interventions de FPT, aucune supplémentation analgésique peropératoire et aucune conversion en AG n’ont été nécessaires.

Les patients ont été généralement opérés dans un délai de 12 (5-36) heures après leurs admission aux urgences soit un délai très optimal par rapport à celui préconisé dans la littérature (24-72 heures) [4, 5]. La fixation précoce de la FPT étant l’un des facteurs principaux impliqués dans la réduction significative de la morbi-mortalité associée à ce type de lésion [4, 5]. Les causes de retard à l’intervention étaient généralement; le délai requis pour une exploration (cardiovasculaire, respiratoire…), la correction d’un désordre métabolique, la nécessité d’une séance de dialyse et la discussion avec l’entourage. L’objectif recherché chez les patients de cette série était surtout de préserver une stabilité hémodynamique et respiratoire afin d’éviter toutes éventuelles décompensations des pathologies sous-jacentes. Vingt-huit patients (93,3%) ont gardé un profil hémodynamique globalement stable, l’hypotension légère et la diminution de la FC observées chez quelques patients après réalisation du BLCS étaient non significatives (p > 0,05), elles s’expliqueraient très probablement par la sédation de la douleur obtenue après installation des blocs nerveux.

L’hypotension prononcée enregistrée chez 2 patients (6,6%) s’expliquerait probablement chez le premier par une éventuelle extension péridurale des AL qui aurait induit un bloc sympathique bilatéral, et chez le deuxième par une hypovolémie infraclinique qui se serait démasquée par le bloc sympathique (patient était cachectique et déshydraté à cause d’une infection respiratoire). Ces chutes tensionnelles étaient néanmoins facilement jugulées par des doses relativement faibles de vasopresseur. Par ailleurs, aucune complication d’ordre respiratoire n’a été enregistrée. L’ALR-PN n’affecte que très peu l’état hémodynamique. Le bloc sympathique résultant du BCLS est généralement inférieur à celui observé après une RA en raison de son caractère unilatéral, il offre en conséquence une meilleure stabilité hémodynamique [10]. Toutes les publications s’accordent sur l’intérêt majeur du BCLS chez les patients graves qui sont très sensibles aux variations hémodynamiques peri-opératoires [10-15, 23, 24]. Cet avantage précieux nous a encouragées à opérer nos patients dans un délai très optimal sans chercher à trop pousser les investigations complémentaires ou ajuster les traitements poursuivis.

Le premier besoin en antalgiques postopératoires était exprimé en moyenne après 8 heures chez la majorité des patients. Ces résultats sont comparables à ceux des études précédentes [10, 12, 15, 21]. L’ALR-PN a démontré depuis longtemps sa supériorité aux autres techniques anesthésiques en matière d’analgésie postopératoire. Le control efficace de la douleur peri-opératoire est d’une importance capitale chez les patients à haut risque anesthésique. En effet ceci a permis une réduction significative des besoins en antalgiques postopératoires en particulier les morphiniques et les anti-inflammatoires épargnant ainsi leurs effets secondaires qui peuvent être néfastes chez une telle population (dépression respiratoire, confusion mentale, saignements gastro-intestinaux, insuffisance rénal…etc.) [25]. De plus cette analgésie suffisante obtenue durant l’intervention et plusieurs heures en postopératoire aurait contribué à la réduction de l’avènement des troubles cognitifs postopératoires en particulier chez les personnes âgées. En effet, certaines études ont apporté des preuves sur les effets de la douleur dans la précipitation de l’avènement des troubles cognitifs chez cette population en cas de FH [26, 27].

Certains auteurs ont utilisé les cathéters perinerveux au plexus lombaire pour prolonger l’analgésie postopératoire [12, 21]. Néanmoins cette option reste discutable car elle n’est pas dénuée d’effets secondaires avec risques de complications potentielles [28, 29]. De plus, elle requière des compétences techniques, des équipements spécialisés, et une surveillance postopératoire rapprochée [30]. De ce fait elle n’a pas été prise en compte dans notre étude. La déambulation postopératoire était très précoce chez la majorité des patients, avec haute satisfaction des patients et de l’équipe chirurgicale. Ceci s’explique par le caractère unilatéral du BCLS qui préserve l’autonomie du membre sain, l’analgésie de qualité obtenue sur plusieurs heures en postopératoire et l’absence de certains effets adverses (NVPO, retentions d’urines, céphalées…etc.). La déambulation précoce contribue à l’amélioration de la réhabilitation postopératoire, prévient l’avènement des complications de décubitus et raccourcis le séjour hospitalier. Le saignement chirurgical et le nombre de culots globulaires transfusés dans cette étude étaient respectivement de 200 ± 50ml (100-700) et 0.5 unités (0-3). Celui rapporté en littérature avec les autres techniques anesthésiques est de 68-583ml [31]. Certaines études ont rapporté des effets positifs du BLP dans la réduction significative des pertes sanguines peropératoires en particulier lors des interventions d’arthroplastie totale de la hanche [32-34].

Le relâchement musculaire peropératoire en particulier dans les régions de forte densité musculaire comme la hanche est d’une importance capitale pour réduire le foyer fracturaire et faciliter les manipulations chirurgicales. Chez la majorité de nos patients il a été jugé optimal par les chirurgiens. Dans les études antérieures le relâchement musculaire obtenu grâce au BCLS a été jugé comparable à celui obtenus par la RA [10, 34]. L’intérêt du BCLS doit être analysé en tenant compte aussi de certains inconvénients et effets adverses nécessitant une évaluation stricte de la balance bénéfice/risque de cette technique. En effet la nécessité de réaliser deux injections douloureuses et la longue durée requise pour la procédure constituent souvent des facteurs limitants et peuvent augmenter l’inconfort des patients. Entre des mains non expérimentées la réalisation du BCLS pourrait être techniquement difficile et le potentiel d’échec devient plus important [10, 35]. Le délai pour la réalisation du BCLS dans notre étude était de 15,20 ± 3,45 minutes (8-25), ce qui s’accorde avec les études précédentes [10, 36]. L’inconfort des patients pendant la réalisation des blocs a été observé chez 4 patients (25%) ce qui est comparable avec les données de la littérature [35]. L’explication de la technique anesthésique, l’adhésion préalable des patients, associés à une sédation de confort nous ont permis de contourner ce problème.

Les volumes d’AL nécessaires aux deux blocs nerveux lombaire et sciatique sont beaucoup plus importants par rapport à la RA ce qui pourrait avoir un potentiel de toxicité supérieur. Néanmoins, les études évaluant la pharmacocinétique de la bupivacaine et de la lidocaine après un BLP avec et sans BS suggèrent que l’absorption de ces AL est généralement lente et les taux sériques produits sont par conséquence sécuritaires [37, 38]. Jusqu’à présent, il y a encore peu de données concluantes en littérature décrivant la posologie optimale pour BLP et le BSP. Nous avons utilisé un volume de 20ml d’AL constitué d’un mélange 50/50 de bupivacaine-lidocaine pour chaque bloc plexique. La bupivacaine a été choisie pour augmenter la durée de l’analgésie et la lidocaine a été ajoutée pour réduire le délai d’installation des blocs nerveux. Aucun cas de toxicité liée aux AL n’a été noté. Cependant, nous ne pouvons pas exclure la possibilité que la taille de la population n’ait été pas assez large pour détecter cette complication rare. La littérature ne rapportant qu’une incidence entre 3,9/10000 et 11/10000 des réactions systémiques aux AL [39].

Aucun cas de neuropathie périphérique n’a été souligné dans cette étude probablement à cause du respect strict des précautions, la réalisation des blocs nerveux par le même operateur, et l’effectif faible de l’étude. Une complication spécifique au BLP qui devrait être soulignée qui est l’extension péridurale des AL (± intrathécale) dont l’incidence varie selon les approches (pouvant aller jusqu’à 16%) [40]. Cette complication a été observée chez un seul patient (3,3%) dans notre étude, et y était responsable d’une hypotension prononcée mais rapidement jugulée par l’éphédrine. Le mécanisme de cette complication reste insuffisamment élucidé, plusieurs théories ont été avancées: une aiguille insérée ou dirigée trop médialement pourrait entraîner une injection directe des AL dans l’espace péridural (ou dans le foramen intervertébral, la gaine dure-mérienne des racines nerveuses ou même dans le canal médullaire), une injection forcée ou trop rapide des AL, l’utilisation de grandes quantités d’AL, les déformations vertébrales etc. [41-44]. De ce fait certaines précautions ont été recommandées durant la pratique du BLP: l’insertion de l’aiguille devrait être perpendiculaire à la peau et la profondeur devrait être guidée par la position de l’apophyse transverse de L4 (15-20mm au maximum au-delà de ce repère) [17].

Une contraction des muscles adducteurs ou de type sciatique indique une position trop médiale de l’aiguille (elle devrait être retirée et réorientée en direction légèrement plus latérale jusqu’à l’obtention de la contraction du muscle quadriceps) [45]. Cette réponse devrait disparaitre avec une intensité du courant <0,35 mA (au cas contraire l’aiguille serait à l’intérieur de la gaine dure-mérienne entourant les racines nerveuses augmentant le risque de diffusion des AL dans l’espace péridural) [45]. De plus, il est recommandé d’utiliser l’échoguidage (± couplé à la neurostimulation) surtout chez les patients avec anatomie difficile ou sous traitements antithrombotiques pour améliorer la précision et prévenir les risques de complications ainsi que pour réduire les volumes injectés des AL [46]. Le taux de mortalité à 30 jours dans notre étude était de 3% (1 seul patient) ce qui est très faible par rapport aux données publiées en littérature (17,4%) [20], et si l’on prend en compte le type de la population étudiée. Ceci s’expliquerait probablement par le délai précoce de l’intervention chirurgicale, la surveillance periopératoire rapprochée, la déambulation précoce et l’absence d’effets adverses rencontrés avec les autres techniques anesthésiques.

A notre connaissance les études ayant exploré le BCLS pour les interventions de fixation de la FPT en particuliers chez les patients à haut risque anesthésique sont très rares. En 2018, Neou *et al*. [36] ont publié une étude prospective sur 50 patients comparant le bloc combiné du compartiment psoas (équivalent du BLP) et le BS à la RA pour les interventions de FPT, confirmant l’efficacité anesthésique du bloc combiné et démontrant sa supériorité par rapport à la RA en matière de stabilité hémodynamique chez les patients avec un statut cardiovasculaire précaire. Plus tôt deux autres études ont traités le BCLS et son intérêt pour toutes les FH, l’une était de type prospectif comparatif (avec la RA) publiée en 2000 par De Visme *et al*. [10] concluant que les deux techniques fournissaient une anesthésie efficace équivalente, et peuvent occasionner avec les mêmes proportions une hypotension marquée en particulier chez les patients de plus de 85 ans. L’autre étude était de type rétrospectif publiée en 2015 par Petchara *et al*. [21] ayant traité le BCLS chez 70 patients confirmant son profil d’efficacité et de sécurité chez les patients gériatriques à haut risque anesthésique. Le reste des publications sont généralement des cas isolés ou des séries de cas de faibles effectifs ayant utilisé efficacement le BCLS pour les FH chez les patients à haut risque anesthésique [11-14].

Cette étude a quelques limites à signaler. Tout d’abord, l’effectif étroit qui demeure très insuffisant pour permettre de tirer des conclusions quant à la faisabilité et la sécurité effectives de cette technique anesthésique. Ensuite l’absence de comparaison à une autre technique de référence telle que la RA ou l’AG. Et enfin les chirurgiens (et infirmiers anesthésistes) dans cette étude étaient rompu aux techniques d’ALR-PN et aux conditions opératoires sous BCLS, ce qui ne reflète pas réellement les conditions de routine surtout pour les acteurs peu formés à ces techniques.

## Conclusion

Ce travail illustre l’intérêt majeur du BCLS comme alternative anesthésique pour les interventions urgentes de FPT chez les patients à haut risque anesthésique: délais opératoires réduits, efficacité anesthésique, stabilité hémodynamique et respiratoire periopératoire, relâchement musculaire adéquat, absence de complications inhérents à l’AG et l’APM (nausées-vomissements, rétentions d’urines, hypotensions, céphalées etc.), passage rapide en SSPI, analgésie postopératoire très efficace avec épargne morphinique considérable, déambulation précoce et enfin une forte satisfaction des patients et de l’équipe chirurgicale. Néanmoins, l’intérêt du BCLS doit être analysé en tenant compte aussi de certains effets adverses qui nécessitent une évaluation stricte de la balance bénéfice/risque. Des études complémentaires à larges effectifs s’avèrent nécessaires pour confirmer nos résultats.

### Etat des connaissances sur le sujet

- Les fractures pertrochantériennes sont très fréquentes et constituent l’un des principaux problèmes de santé à travers le monde du fait de leur morbi-mortalité très élevée;
- L’anesthésie pour la chirurgie urgente de ces fractures chez les patients à haut risque anesthésique représente souvent un véritable challenge pour les praticiens en vue du risque periopératoire majeur;
- La rachianesthésie a été depuis longtemps privilégiée par la plupart des opérateurs comme technique anesthésique de choix pour ces interventions car elle concilie les impératifs chirurgicaux et anesthésiques.

### Contribution de notre étude à la connaissance

- Entre des mains expérimentées le bibloc lombaire et sciatique plexique constitue une alternative anesthésique prometteuse pour les interventions urgentes de fracture pertrochantérienne chez les patients à haut risque anesthésique.

## References

[1] Mavrogenis AF, Panagopoulos GN, Megaloikonomos PD, Igoumenou VG, Galanopoulos I, Vottis CT (2016). Complications After Hip Nailing for Fractures. Orthopedics.

[2] Waast D, Touraine D, Wessely L, Ropars M, Coipeau P, Perrier C (2007). Pertrochanteric fractures in elderly subjects aged over 75. Rev Chir Orthop Reparatrice Appar Mot.

[3] Johnell O, Kanis JA (2004). An estimate of the worldwide prevalence, mortality and disability associated with hip fracture. Osteoporos Int.

[4] Roche JJ, Wenn RT, Sahota O, Moran CG (2005). Effect of comorbidities and postoperative complications on mortality after hip fracture in elderly people: Prospective Observational Cohort Study. BMJ.

[5] Simunovic N, Devereaux PJ, Sprague S, Guyatt GH, Schemitsch E, Debeer J (2010). Effect of early surgery after hip fracture on mortality and complications: systematic review and meta-analysis. CMAJ.

[6] Rodgers A, Walker N, Schug S, McKee A, Kehlet H, van Zundert A (2000). Reduction of postoperative mortality and morbidity with epidural or spinal anaesthesia: results from overview of randomised trials. BMJ.

[7] Parker MJ, Handoll HH, Griffiths R (2004). Anaesthesia for hip fracture surgery in adults. Cochrane Database Syst Rev.

[8] Urwin SC, Parker MJ, Griffiths R (2000). General versus regional anaesthesia for hip fracture surgery: a meta-analysis of randomized trials. Br J Anaesth.

[9] Rashid RH, Shah AA, Shakoor A, Noordin S (2013). Hip Fracture Surgery: Does Type of Anesthesia Matter?. Biomed Res Int.

[10] de Visme V, Picart F, Le Jouan R, Legrand A, Savry C, Morin V (2000). Combined lumbar and sacral plexus block compared with plain bupivacaine spinal anesthesia for hip fractures in the elderly. Reg Anesth Pain Med.

[11] Chia N, Low TC, Poon KH (2002). Peripheral nerve blocks for lower limb surgery—a choice anaesthetic technique for patients with a recent myocardial infarction?. Singapore Med J.

[12] Ho AM, Karmakar MK (2002). Combined paravertebral lumbar plexus and parasacral sciatic nerve block for reduction of hip fracture in a patient with severe aortic stenosis. Can J Anaesth.

[13] Asao Y, Higuchi T, Tsubaki N, Shimoda Y (2005). Combined paravertebral lumbar plexus and parasacral sciatic nerve block for reduction of hip fracture in four patients with severe heart failure. Masui.

[14] Eker HE, Kocum A, Kocum T, Turkoz A, Arslan G Severe Aortic Stenosis: Combined Lumbar Plexus, Sciatic and Iliohypogastric Nerve Block with 0.25% Levobupivacaine for Reduction of Hip Fracture. Internet J Anesthesiol.

[15] Laguillo Cadenas JL, Martínez Navas A, Ortiz de la Tabla González R, Ramos Curado P, Echevarría Moreno M (2009). Bloqueo combinado del plexo lumbar por vía posterior y plexo sacro para tratamiento quirúrgico urgente de la fractura de cadera. Rev Esp Anestesiol Reanim.

[16] Klein SM, Pietrobon R, Nielsen KC, Warner DS, Greengrass RA, Steele SM (2002). Peripheral nerve blockade with long-acting local anesthetics: a survey of the Society for Ambulatory Anesthesia. Anesth Analg.

[17] Capdevila X, Macaire P, Dadure C, Choquet O, Biboulet Ph, Ryckwaert Y (2002). Continuous psoas compartment block for postoperative analgesia after total hip arthroplasty: New landmarks, technical guidelines, and clinical evaluation. Anesth analg.

[18] Mansour NY (1993). Reevaluating the sciatic nerve block: another landmark for consideration. Reg Anesth.

[19] Bromage PR (1978). Epidural Analgesia.

[20] Karaca S, Ayhan E, Kesmezacar H, Uysal O (2012). Hip fracture mortality: is it affected by anesthesia techniques?. Anesthesiol Res Pract.

[21] Petchara S, Paphon S, Vanlapa A, Boontikar P, Disya K (2015). Combined Lumbar Sacral Plexus Block in High Surgical Risk Geriatric Patients undergoing Early Hip Fracture Surgery. Malays Orthop J.

[22] Birnbaum K, Prescher A, Hessler S, Heller KD (1997). The sensory innervation of the hip joint-an anatomical study. Surg Radiol Anat.

[23] Naja Z, el Hassan MJ, Khatib H, Ziade MF, Lönnqvist PA (2000). Combined sciatic-paravertebral nerve block vs general anaesthesia for fractured hip of the elderly. Middle East J Anaesthesiol.

[24] Fanelli G, Casati A, Aldegheri G, Beccaria P, Berti M, Leoni A (1998). Cardiovascular effects of two different regional anaesthetic techniques for unilateral leg surgery. Acta Anaesthesiol Scand.

[25] Morrison RS, Magaziner J, McLaughlin MA, Orosz G, Silberzweig SB, Koval KJ (2003). The impact of post-operative pain on outcomes following hip fracture. Pain.

[26] Marcantonio ER, Flacker JM, Wright RJ, Resnick NM (2001). Reducing delirium after hip fracture: a randomized trial. J Am Geriatr Soc.

[27] Milisen K, Foreman MD, Abraham IL, De Geest S, Godderis J, Vandermeulen E (2001). A nurse-led interdisciplinary intervention program for delirium in elderly hip fracture patients. J Am Geriatr Soc.

[28] Pousman R, Mansoor Z, Sciard D (2003). Total Spinal Anaesthetic after Continuous posterior Lumbar Plexus Block. Anesthesiology.

[29] Ben-David B, Joshi R, Chelly J (2003). Sciatic Nerve Palsy after Total Hip Arthroplasty in a Patient Receiving Continuous Lumbar Plexus Block. Anesth Analg.

[30] Hirst GC, Lang SA, Dust WN, Cassidy JD, Yip RW (1996). Femoral nerve block: Single injection versus continuous infusion for total knee arthroplasty. Reg Anesth.

[31] Luger TJ, Kammerlander C, Gosch M, Luger MF, Kammerlander-Knauer U, Roth T (2010). Neuroaxial versus general anaesthesia in geriatric patients for hip fracture surgery: does it matter?. Osteoporos Int.

[32] Twyman R, Kirwan T, Fennelly M (1990). Blood loss reduced during hip arthroplasty by lumbar plexus block. J Bone Joint Surg Br.

[33] Stevens RD, Van Gessel E, Flory N, Fournier R, Gamulin Z (2000). Lumbar plexus block reduces pain and blood loss associated with total hip arthroplasty. Anesthesiology.

[34] Buckenmaier CC, Xenos JS, Nilsen SM (2002). Lumbar Plexus Block with Perineural Catheter and Sciatic Nerve Block for Total Hip Arthroplasty. J Arthroplasty.

[35] Fanelli G, Casati A, Garancini P, Torri G (1999). Nerve stimulator and multiple injection technique for upper and lower limb blockade: failure rate, patient acceptance, and neurologic complications. Study Group on regional Anesthesia. Anesth Analg.

[36] Neou E, Fyrfiris N, Katounis C, Barkas K, Tsailas PG (2018). Combination of Psoas compartment and sciatic nerve blocks vs spinal block for Intertrochanteric Fracture Surgery. Middle East J Anaesthesiol.

[37] Farny J, Girard M, Drolet P (1994). Posterior approach to the lumbar plexus combined with a sciatic nerve blockusing lidocaine. Can J Anaesth.

[38] Odoom JA, Zuurmond WWA, Sih IL, Bovill J, Osterlof G, Oosting HV (1986). Plasma bupivacaine concentrations following psoas compartment block. Anaesthesia.

[39] Auroy Y, Narchi P, Messiah A, Litt L, Rouvier B, Samii K (1997). Serious complications related to regional anesthesia: results of a prospective survey in France. Anesthesiology.

[40] Parkinson SK, Mueller JB, Little WL, Bailey SL (1989). Extent of blockade with various approaches to the lumbar plexus. Anesth Analg.

[41] Mannion S, O’Callaghan S, Walsh M, Murphy DB, Shorten GD (2005). In with the new, out with the old? Comparison of two approaches for psoas compartment block. Anesth Analg.

[42] De Biasi P, Lupescu R, Burgun G, Lascurain P, Gaertner E (2003). Continuous lumbar plexus block: use of radiography to determine catheter tip location. Reg Anesth Pain Med.

[43] Mannion S (2004). Epidural spread depends on the approach used for posterior lumbar plexus block. Can J Anaesth.

[44] Gadsden JC, Lindenmuth DM, Hadzic A, Xu D, Somasundarum L, Flisinski KA (2008). Lumbar Plexus Block Using High-pressure Injection Leads to Contralateral and Epidural Spread. Anesthesiology.

[45] Destrubé M, Guillou N, Orain C, Chaillou M, Ecoffey C (2007). Psoas compartment block with general anaesthesia: descriptive study of 93 cases. Ann Fr Anesth Reanim.

[46] Karmakar MK, Ho AM, Li X, Kwok WH, Tsang K, Ngan Kee WD (2008). Ultrasound-guided lumbar plexus block through the acoustic window of the lumbar ultrasound trident. Br J Anaesth.

